# ERA’s ABCDE framework for kidney disease prevention: turning the WHO kidney health resolution into action

**DOI:** 10.1093/ndt/gfaf198

**Published:** 2026-01-08

**Authors:** European Renal Association (ERA) Council:, Roser Torra, Dimitrios S Goumenos, Mustafa Arici, Alberto Ortiz, Marcin Adamczak, Kathrin Eller, Ana Carina Ferreira, Giuseppe Grandaliano, Kitty Jager, Jennifer Lees, Vassilios Liakopoulos, Roberto Minutolo, Siren Sezer, ERA Senior Staff:, Federico Torres, Laura Azzolini, Monica Fontana

**Keywords:** albuminuria, chronic kidney disease, prevention of CKD, pre-CKD

## Abstract

In 2025, the World Health Assembly of the World Health Organization (WHO) adopted a resolution on reducing the burden of noncommunicable diseases (NCDs) by promoting kidney health and strengthening the prevention and control of kidney disease. Following the WHO resolution, the United Nations (UN) included kidney health in its 2025 Political Declaration on NCDs. These measures are a clear response to the growing burden of kidney diseases. This achievement for kidney health was facilitated by years of effort by multiple stakeholders and decision-makers, including nephrology associations, particularly the International Society of Nephrology, the European Renal Association (ERA) and the American Society of Nephrology, gathering evidence on the growing burden of chronic kidney disease (CKD), raising awareness of this burden, advancing research and innovation, and adopting scientific and policy recommendations for the early detection, prevention and treatment of CKD. The WHO and UN measures add kidney disease to a list of major NCDs (e.g. cancer, cardiovascular diseases, diabetes, respiratory diseases) that should be prioritized by healthcare systems. The kidney health resolution is fully aligned with the ERA’s activities and recommendations, as well as with the 2025 KDIGO document on the prevention of CKD and maintenance of kidney health. This novel preventive approach has been tried and tested for other conditions, such as cardiovascular disease, the age-adjusted mortality of which is falling dramatically, compared with the equally dramatic increase in CKD mortality. The next step would be to define an actionable condition of very high risk of CKD, that may be termed pre-CKD. This should be complemented by programs for the early diagnosis and treatment of CKD, such as the one promoted by ERA’s ‘Protect Your Kidneys, Protect Your Future’ campaign which emphasizes the need to know and treat the ABCDE numbers (Albuminuria, Blood pressure, Cholesterol, Diabetes, Estimated glomerular filtration rate) to improve cardiovascular–kidney–metabolic health.

## BACKGROUND

On 23 May 2025, the 78th World Health Assembly of the World Health Organization (WHO) adopted a resolution on reducing the burden of noncommunicable diseases (NCDs) by promoting kidney health and strengthening the prevention and control of kidney disease [1]. Among other topics, the resolution urges Member States, in accordance with their national context and priorities, to invest in health systems to integrate prevention, early detection and management of kidney disease into national health policies and inclusion of kidney management into universal health coverage benefit package, including the universal access to kidney replacement therapy (KRT) (Box 1). It further requests the WHO Director General to advance kidney disease as an NCD of increasing global priority. In 2024, the WHO listed four major NCDs as causes of death and disability (cancer, cardiovascular diseases, diabetes and respiratory diseases [2]). While diabetic kidney disease was under the umbrella of ‘diabetes’, other kidney diseases were not. The 2025 WHO kidney health resolution further calls for the strengthening and the integration of the kidney disease monitoring—including burden, access to care, quality of care, and morbidity and mortality outcomes—into national health information systems to inform and shape policy decisions and guide research. On 25 September 2025, the 4th United Nations High Level Meeting on NCDs and Mental Health also added kidney disease to the list of NCDs to be prioritized for prevention and control in the next decade, in order to reduce the overall burden of NCDs on individuals and societies. While the WHO resolution urges Member States to take specific actions to improve awareness, prevention, and management of kidney diseases—and expects WHO to provide guidance and technical support—the prioritization of kidney diseases in the UN Political Declaration on NCDs carries greater political weight and commitment by UN Member States (including almost all European countries). UN Political Declarations are endorsed at the heads of state and government level, and are therefore expected to shape NCD agendas, priorities, funding and programmes for the next decade.

These measures, adopted by leading international organizations, are a clear response to the growing burden of kidney diseases and are expected to guide political and health authorities throughout the world in strengthening their responses. This achievement in kidney health is the result of years of dedicated efforts by multiple stakeholder organizations and decision-makers. Nephrology associations—most notably the International Society of Nephrology, for its leading advocacy at the international level,

Box 1. **Reducing the burden of NCDs through the promotion of kidney health and strengthening prevention and control of kidney disease.**

Key messages

• Prioritizing kidney health

• Integration of kidney health into national NCD strategies

• Promoting awareness through education campaigns about kidney disease risk factors and preventive measures

• Promoting healthy lifestyle for kidney health

• Strengthening prevention and early detection, and treatment of kidney disease, particularly in high-risk populations

• Improving access to affordable and quality treatment, including kidney replacement therapies, especially in low- and middle-income countries

• Strengthening health systems to effectively manage kidney disease across the continuum of care

• Multi-sectoral collaboration, including healthcare, research and policy, to address the multifaceted nature of kidney disease

• Tracking the burden of kidney disease

but also the European Renal Association (ERA), the American Society of Nephrology and the Sociedad Latinoamericana de Nefrología e Hipertensión—together with the European Kidney Health Alliance (EKHA) and the other signatories of the letters to the WHO and UN (Supplementary data) have been instrumental in raising awareness of the growing burden of kidney diseases, while also advancing scientific and policy recommendations aimed at enhancing the prevention and management of these conditions [3]. In the case of ERA, its vision and scientific and educational activities are fully aligned with the WHO and UN goals and recommendations. The ERA, both independently and as part of EKHA, is ready to continue working with the European Union (EU) and national policymakers to develop and implement medical and policy solutions that improve prevention and ensure accessible and innovative care across Europe [4].

**Figure 1:**
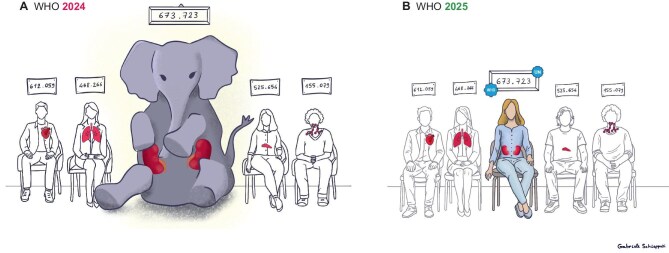
CKD: in 2025, the elephant in the room has been recognized a major human health issue requiring urgent attention. (**A**) In 2024, CKD was the elephant and shared the room with other prioritized non-communicable diseases (NCD) [2]. The number above each condition represents the global prevalence in thousands according to the Institute for Health Metrics and Evaluation (IHME) and the Global Burden of Disease (GBD) study [61]. Diseases from left to right: heart diseases, stroke, pulmonary diseases, CKD, diabetes mellitus, and cancer. Note that the IHME global prevalence of CKD underestimates the 850 million previous estimate by Nephrology experts for all kidney diseases combined. (**B**) In 2025, the WHO and UN considered kidney disease as prioritized NCD, thus humanizing the burden of CKD, the most prevalent of kidney diseases. Prevalence data are from 2021. Updated GBD data for CKD will be made public on Sunday, 12 October 2025, alongside the publication of a special issue of *The Lancet*.

Recognizing kidney disease as one of the top NCDs to be prioritized globally was long overdue, as it represented the ‘elephant in the room’ given its prevalence (850 million) and growing impact on global deaths, a challenge that surpasses some of the previously prioritized NCDs [5]. In essence, the WHO and UN action has put a human face on the ‘elephant’ (Fig. 1). Chronic kidney disease (CKD) is the most common kidney disease and the fastest growing cause of death in Europe [6]. It is projected to become the third leading cause of death in Western Europe and the fifth in the world by 2050 [7]. While mortality from CKD is also growing fast in Central Europe (projected 23% increase in age-standardized death rate between 2022 and 2050) and Eastern Europe (projected 40% increase), these regions are still spared the full negative impact of CKD, likely because of shorter life expectancy (Eastern Europe 78 years, Central Europe 81 years, Western Europe 84 years), with older age being one of the main risk factors for CKD. However, in all three European regions, age-adjusted mortality from ischaemic heart disease and stroke is forecasted to decrease by 48% to 52% from 2022 to 2050, illustrating the beneficial impact of ongoing preventive strategies. Primary prevention of atherosclerotic cardiovascular and cerebrovascular diseases is widely implemented by treating hypercholesterolemia and hypertension, two preventive strategies enshrined in guidelines for decades [8]. In contrast, the concept of actively preventing CKD (primary prevention, i.e. avoiding the development of CKD by intervening at a pre-CKD stage) has been missing from major guidelines. The KDIGO 2024 clinical practice guideline for the evaluation and management of CKD focuses on events occurring AFTER CKD diagnosis [9]. This contrasts with the over 100-page 2021 European Society of Cardiology (ESC) Guidelines on cardiovascular disease prevention in clinical practice [10] or specific chapters on diabetes prevention in diabetes guidelines [11]. Only in 2025 did the KDIGO Controversies Conference address the issue of maintaining kidney health in a publication [12]. Furthermore, while the KDIGO guideline on CKD provides a framework for the early diagnosis of CKD based on evidence of kidney injury using, for example, the criterion of a urinary albumin–creatinine ratio (UACR) above 30 mg/g, albuminuria testing is not widely implemented outside the diabetes mellitus context, and even for diabetes, UACR is only assessed in around 50% of patients [13]. The low uptake of albuminuria testing precludes widespread diagnoses of early-stage CKD, when kidney function remains above the glomerular filtration rate (GFR) threshold used to diagnose CKD (60 mL/min/1.73 m^2^), and where treatment may delay the need for KRT by nearly three decades [14]. From a policy perspective, giving greater room to prevention and detection of CKD is more cost effective and can substantially reduce the financial strain on health systems and patients. Furthermore, providing KRT, especially dialysis, is associated with high costs for society and for individuals (e.g. related to loss of work opportunities) as well as to a dramatically negative environmental impact [15, 16]. In the absence of a holistic preventive strategy anchored to widespread access to early diagnosis and treatment, it is unlikely that the burden of CKD will decrease in the foreseeable future.

The WHO kidney resolution acknowledges that Member States may have different resources, national contexts and priorities [1]. CKD is forecast to become the most common cause of death by 2050 in Guatemala—the country sponsoring the declaration with Thailand, which submitted the resolution, as Guatemala was not on the executive board of the WHO in 2025—as well as in other Central American countries such as Panama, Costa Rica, El Salvador and Nicaragua [7]. For these countries, CKD is likely the main health priority.

In high-income countries, CKD is projected to become the third leading cause of death by 2050. Those with longer life expectancy, such as Western Europe or Japan, will be hit hardest and should lead the way in addressing this growing burden [17]. High-income countries may have better access to resources, enabling them to address key unmet needs in kidney health through holistic preventive strategies and early diagnosis programmes.

The USA, for example, has a threefold higher incidence of kidney failure requiring KRT than Europe, along with higher mortality on KRT [18]. However, its healthcare system is largely oriented towards treating advanced diseases, which has resulted in one of the lowest life expectancies among high-income countries.

By contrast, Europe is well positioned to lead the change in maintaining kidney health. Despite their differences, European countries—including EU Member States—possess robust healthcare systems, clinical expertise and public health infrastructures capable of driving early diagnosis of CKD, preserving or restoring kidney function, and identifying and treating people at high risk in a preventive manner. Nevertheless, regional differences persist: preventive strategies are more widely implemented in Western Europe, while Central/Eastern Europe continues to face barriers to early diagnosis and treatment. With more harmonized approaches, these disparities will be reduced. By strengthening primary care capacities, expanding screening programmes and promoting public awareness, national health authorities can take decisive action to reduce the burden of CKD. Individual countries should also address inequalities in access to conservative care and to different forms of KRT, giving prioritization to kidney transplantation [19–22].

The EU should play a key role in supporting and complementing these national efforts, for example, by providing funding for research and innovation—including support for registries—fostering pan-European research collaborations, harmonizing guidelines, standards of care and best practices based on scientific evidence endorsed by the ERA, KDIGO and other scientific societies, as well as facilitating knowledge exchange. In addition, EU-level coordination and policy guidance, in line with WHO (and UN) recommendations, can help ensure coherent prevention strategies, so that Member States’ actions are mutually reinforcing and kidney health remains a shared priority across Europe.

## ERA AND CKD PREVENTION: ALIGNMENT WITH THE WHO RESOLUTION ON KIDNEY HEALTH

The ERA Registry provides summaries of inequalities in CKD epidemiology and access to KRT across Europe, which can be used for benchmarking discussions with local health authorities.

However, a major information gap still exists regarding the epidemiology of early CKD—especially stages G1 and G2—throughout the life cycle, due in part to the suboptimal uptake of albuminuria testing and to limited data from underserved communities. The epidemiology of CKD is also evolving, influenced by population ageing, increasing rates of overweight/obesity, diabetes and hypertension, as well as a shifting genetic landscape due to migration from Sub-Saharan Africa, Asia and Latin America. For example, APOL1 risk variants that predispose individuals to CKD are highly prevalent in Sub-Saharan Africa, while a predisposition to severe immune-mediated nephropathies is seen in Latin American individuals with Native American ancestry [23, 24].

Given these changes, the overall epidemiology of CKD in Europe should be periodically re-evaluated and updated.

Beyond updating the numbers on the burden of CKD needed by health authorities to plan care, other major unmet needs include the unawareness of the population, healthcare workers, and health authorities of the concept and burden of CKD, the lack of a comprehensive preventive approach and suboptimal approaches to early diagnosis and aetiology workup.

**Figure 2:**
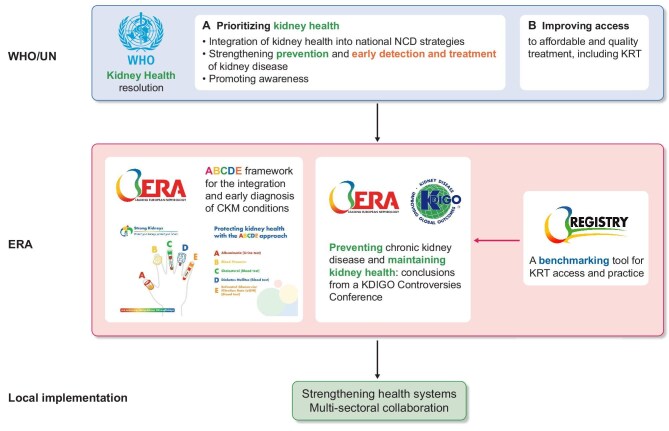
2025 Kidney health resolution by the WHO supported by the UN: alignment with ERA vision and structures and need for local implementation.

The WHO Resolution and the UN Political Declaration prioritizing kidney health will help all stakeholders in kidney disease to advocate for these issues, ensuring that the ‘elephant in the room’ is visible to all (Fig. 1). ERA’s activities and structures are fully aligned with the vision of the WHO and UN (Fig. 2) and as such, ERA is committed to working with decision-makers and stakeholders to put their recommendations into action.

## INCREASING AWARENESS ABOUT KIDNEY DISEASE

To increase awareness of kidney disease, the ERA launched the ‘Protect Your Kidneys, Protect Your Future’ campaign in 2024, targeting the healthcare community, policymakers and the general public [25]. A key element of this campaign is the ABCDE framework [26] for the holistic assessment of cardiovascular–kidney–metabolic (CKM) health [27–30]. The CKM syndrome was recently conceptualized by the American Heart Association to emphasize the close links between overweight-obesity, type 2 diabetes mellitus, kidney health and cardiovascular health [31]. Here, ABCDE stands for Albuminuria, Blood pressure, Cholesterol, Diabetes (i.e. glycaemia) and Estimated glomerular filtration rate [27–30] (Fig. 2), i.e. for one urine test, one physical examination feature and three blood analytes that allow the correct fitting of individuals in the CKM spectrum requiring specific drug interventions for the primary prevention of CVD, according to the 2021 ESC guidelines [32]. In other words, it summarizes the CKM assessments that should be provided by the healthcare system, as other key elements to assess CKM health that can be self-assessed such as body mass index, smoking status and lifestyle.

## ADDRESSING THE LACK OF A COMPREHENSIVE PREVENTIVE APPROACH

**Figure 3:**
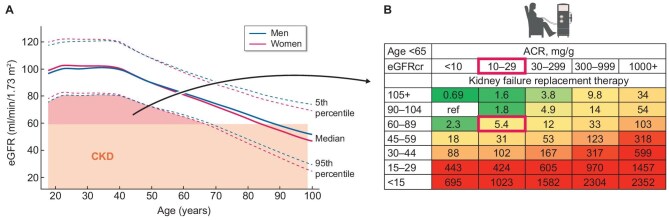
Using eGFR values in the healthy general population to identify individuals potentially at high risk of CKD who may benefit from interventions or participation in clinical trials. (**A**) Age- and sex-specific reference values of eGFR for healthy European adults. ‘Healthy’ is defined as no known risk factors for accelerated aging beyond genetics and age [40]. Note that in people younger than 65 years old, there is an eGFR gap between the threshold that allows by itself to diagnose CKD (<60 mL/min/1.73 m^2^) and the 95% confidence interval of eGFR values found in healthy people. This gap identified people with eGFR which is lower than expected for age yet does not lead to a diagnosis of CKD. According to current guidelines, these people are not offered any diagnostic tests nor interventions. Despite this, some of them have a high risk of kidney failure. In the future, acknowledging their potential high risk of kidney failure may lead to testing for albuminuria and recommending lifestyle or other interventions. (**B**) Adjusted hazard ratio for the development of kidney failure requiring KRT. Note a 5.4-fold higher risk when eGFR is slightly low and albuminuria slightly high, without any of them meeting thresholds (eGFR <60 mL/min/1.73 m^2^ or UACR >30 mg/g) to diagnose CKD by themselves or in combination [38].

Addressing the lack of a comprehensive preventive approach requires clearly defining populations at very high risk of CKD. These groups may benefit from targeted interventions that go beyond lifestyle changes which are recommended to everyone. Preventing CKD has so far been limited to treating risk factors, such as diabetes and hypertension. However, this is not enough, as evidenced by the increasing burden of CKD. Around 75% of patients on KRT in Europe do not have diabetes or hypertension as the cause of CKD [6, 33]. Moreover, not all treatments for diabetes mellitus are equally effective in preventing CKD: evidence from clinical trials and real-world clinical practice indicates that sodium-glucose cotransporter 2 (SGLT2) inhibitors and glucagon-like peptide-1 receptor agonists (GLP1-RA) can prevent the development of CKD, as compared with other agents [11, 34, 35]. Additionally, there are limits to the extent to which treating hypertension can prevent CKD or its progression. Strict blood pressure control decreases the risk of CVD and mortality, but it does not reduce, and may even increase, the risk of kidney failure [36, 37]. Finally, in 2023, the Writing Group for the CKD Prognosis Consortium identified a subgroup of people who did not meet the standard estimated GFR (eGFR) and UACR diagnostic criteria for CKD but still showed a higher risk of kidney failure (adjusted hazard ratio 5.4 in those under 65 years with GFR 60–89 mL/min/1.73 m^2^ and UACR 10 to 29 mg/g; 2.6 for older individuals, in both cases compared with a reference group consisting of eGFR 90–104 mL/min/1.73 m^2^ and UACR <10 mg/g) (Fig. 3) [38]. They, a subgroup of them or a wider group including them, may represent individuals at very high risk of CKD who may benefit from interventions to prevent CKD. The feasibility of a successful pharmacologic preventive intervention is supported by post-hoc analyses of over 20 000 participants in cardiovascular outcome trials of SGLT2 inhibitors in type 2 diabetes mellitus, in whom the risk of new onset CKD was halved, and also beyond diabetes, since the observed chronic eGFR slopes on SGLT2 inhibitors were around 0 mL/min/min/1.73 m^2^/year for the duration of the trials, i.e. slower than the age-associated loss of eGFR [11]. In 2025, the ERA Registry advanced this aim by defining age- and sex-specific reference values for eGFR in European adults, identifying a gap between the 60 mL/min/1.73 m^2^ threshold to diagnose CKD and the lower limit of the confidence interval of the healthy population eGFR values for younger individuals [39, 40]. People with eGFR values within this gap have lower than expected GFR values for their young age. Although they currently do not fulfil criteria to diagnose CKD or to perform additional testing (e.g. albuminuria, imaging), in a future scenario, they may be candidates for albuminuria testing, which may identify CKD if albuminuria is over 30 mg/g, or a very high risk of CKD (e.g. albuminuria 10-<30 mg/g, lower than expected GFR values) [38] (Fig. 3). These individuals at very high risk of CKD who may benefit from active preventive interventions (e.g. if they have type 2 diabetes mellitus) or participate in clinical trials of CKD prevention. The ERA new initiative, ‘ERA Science Meeting’, will address this unmet need in the near future, dedicating one meeting to a consensus definition of a numbers-based stratification into very high risk of CKD. This condition of very high risk may be termed pre-CKD, as a recognizable name reminiscent of the widely adopted concept of prediabetes [11]. Pre-CKD may represent the most advanced stages of what has been previously labelled the blind spot of the current CKD definition, i.e. the journey from initiation of kidney injury to the loss of around 50% of the functional kidney mass or to the increase of the UACR levels to values 10-fold higher than physiologically normal [41, 42]. Recognizing this blind spot early enough will allow preventive measures to be implemented effectively and halt further damage and burden that may occur due to advanced CKD. After conducting the necessary research and data analysis, it will be possible to assess the applicability of current and emerging treatments in this population.

## IMPROVING EARLY DIAGNOSIS OF CKD

The lack of early diagnosis can be approached by implementing albuminuria testing as a cost-effective method of identifying patients in the early (G1-G2) CKD categories who may benefit from active intervention. In this regard, clinical guidelines should be implemented by expanding albuminuria testing to the 21 CKD risk factors for CKD listed by KDIGO [8, 10], and to older age, as recommended by some local guidelines, like those in Spain, that add age above 60 years to the list [43]. Thus, the most common risk factor for KRT is older age, with over 50% of patients initiating KRT being 65 years or older, and older age is associated with an almost 200-fold higher incidence of KRT [17, 33, 6]. KRT remains a more common outcome than death in older patients with CKD under nephrology care [44]. Acknowledging older age as the main risk factor for kidney failure has consequences, despite being a non-modifiable risk factor. The increased risk associated with age is the basis for universal cancer screening programs (e.g. colorectal, breast, cervix cancer) based on age. An argument can be made that high income European countries should consider and eventually offer universal albuminuria screening in programs potentially linked to colorectal screening programs, an approach that may be more cost-effective than cancer screening programs themselves [45–47]. Indeed, recent analysis support the cost-effectiveness of population-wide screening for CKD from ages 55 to 75 years to improve population health and reduce disparities across social groups [48]. Integration of kidney health into national NCD strategies is key for implementation. In Italy, a legislative initiative will support population-wide CKD screening in this age range for people with additional risk factors [49]. These screening campaigns contribute to increasing the awareness of the population of the conditions being screened for.

The need for early diagnosis and intervention cannot be overemphasized. Early (G1-G2) intervention may delay the need for KRT by up to three decades [13]. Once kidney failure develops, life expectancy can be up to 44 years shorter on dialysis and 22 years shorter for carriers of functioning kidney grafts than in the general European population [50].

## OPTIMIZING THE SEARCH FOR THE CAUSES OF CKD

**Figure 4:**
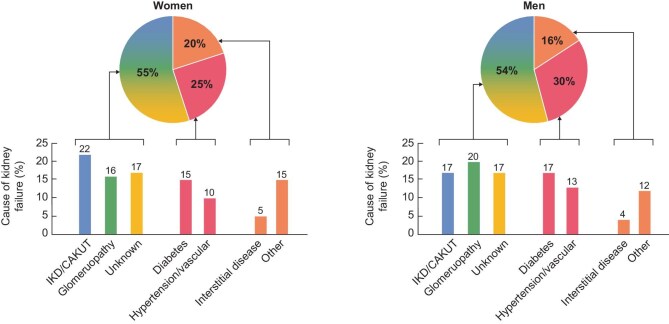
Causes of kidney failure in patients on KRT in the ERA Registry area. Note that the most common causes of kidney failure were rare diseases related to glomerulopathies, inherited and congenital conditions or CKD in which the cause was not identified (over 50% in both men and women). By contrast, common systemic conditions such as diabetes of hypertension/vascular disease only account for roughly one in three patients on KRT at a given moment (prevalent). Figure shows prevalent data for December 2019. The list of causes is completed by interstitial diseases and other rare causes [54].

A suboptimal aetiologic workup is common in CKD [33]. The CKM syndrome emphasizes prevalent conditions linked to CKD for screening. However, in most people with prevalent kidney failure on KRT, the cause is a rare disease, mainly glomerular or genetic, or has not been characterized [33, 51–53] (Fig. 4). These rare conditions will also benefit from the 2025 WHO resolution on rare diseases [54]. Screening for CKD should be tailored to identify this silent majority of people on KRT. Moreover, every effort should be made to determine the cause of CKD, as this may uncover potential treatments, prevent unnecessary interventions, improve kidney transplant outcomes, enable participation in clinical trials, facilitate early diagnosis in family members and provide opportunities for genetic counselling [55, 56]. Genetic kidney disease and congenital anomalies of the kidneys and urinary tract (which may be of genetic origin) are the most common causes of prevalent KRT in women and in people younger than 45 years old [54]. A recent study from Spain found that in roughly one in four patients on KRT younger than 45 years without a clear cause of CKD, genetic testing may be informative [57]. The ‘Genes & Kidney’ Working Group of the ERA recently proposed the term CKD of unexplained cause (CKDx) to reflect cases in which the etiology workup was unable to find a cause, as opposed to cases in which the cause remained unclear after a thorough aetiology workup [58].

This was also true in glomerular diseases, particularly in immunoglobulin A nephropathy (IgAN). IgAN is the most common glomerular cause of kidney failure in Europe [57]. In recent years, new, safer and more specific IgAN therapies have become available, after years of stagnation [59, 60]. However, the low uptake of albuminuria and urinary sediment testing precludes the widespread diagnosis of early-stage IgAN. Late referral to nephrologists and the low accessibility and affordability of kidney biopsies are the other barriers for early diagnosis and effective treatment. In this regard, albuminuria testing is still not reimbursed outside diabetes even in high income European countries with very high incidence of kidney failure requiring KRT, such as Belgium [33]. This represents a key barrier for early diagnosis of CKD. The WHO identification of kidney health as a key priority, independent of diabetes, and its emphasis on rare conditions, may help advocate to achieve reimbursement of low-cost tests for the early identification of kidney conditions that have specific therapeutic approaches.

The next two editions of the ‘ERA Science Meeting’ (2025 and 2026) will provide a roadmap for the implementation of precision medicine in genetic kidney diseases in Europe and a comprehensive approach to glomerular diseases. These two broad categories encompass multiple rare conditions that together account for 37% of men on KRT in Europe (vs 29% for diabetes and hypertension) and 38% of women (vs 24% for diabetes and hypertension) [54].

## CONCLUSION

The 2025 WHO Resolution on Kidney Health, together with the UN High-Level Political Declaration on NCDs, underscores the urgent need to address the rising burden of kidney diseases—now recognized as one of the major global health challenges of the coming decade. Alongside the first KDIGO document on the maintenance of kidney health, they highlight the need for a new approach: one that prioritizes proactive prevention as a complement to the current reactive treatment model. This novel approach has been tried and tested for other conditions, such as cardiovascular disease, whose age-adjusted mortality is falling dramatically (as examples, the 2050 forecast is 41%–44% lower worldwide for cerebrovascular disease and ischaemic heart disease, 48%–52% in Europe), as compared with the equally dramatic increase in CKD mortality (33% higher worldwide by 2050, 23%–40% in Europe) [7].

The ERA strongly welcomes the WHO Resolution and the UN Political Declaration, and is fully aligned with the principle that prevention, together with early diagnosis and treatment of CKD, are essential to reversing its rising burden. It is now time for all clinical, scientific, societal and government stakeholders to work together to place kidney health high on the agenda—and to act before the ‘elephant in the room’ grows even bigger.

The ERA will continue to work closely with its national nephrology societies, policymakers at both the EU and national levels, and other key stakeholders to drive concrete, evidence-based progress in advancing CKD prevention across Europe.

## Supplementary Material

gfaf198_Supplemental_File
