# Supplementary material for: ERA’s ABCDE framework for kidney disease prevention: turning the WHO kidney health resolution into action
Source: Nephrol Dial Transplant. 2026 Jan 8;41(4):793–801. doi: 10.1093/ndt/gfaf198 (PMC13037474; doi:10.1093/ndt/gfaf198)
Supplement: gfaf198_Supplemental_File [file gfaf198_supplemental_file.docx]

**Supplementary data**

**Signatories to the letter on kidney health submitted to the UN** (https://www.era-online.org/wp-content/uploads/2025/07/Open-Letter_2025_HLM-Political-Declaration.pdf).*

- African Association of Nephrology (AFRAN)
- Africa NCDs Network (ANN)
- American Society of Nephrology
- American Society of Pediatric Nephrology
- Asian Pacific Society of Nephrology
- Asian Pediatric Nephrology Association
- Australia and New Zealand Paediatric Nephrology Society
- European Kidney Health Alliance
- European Renal Association
- European Society of Pediatric Nephrology
- Global Patient Alliance for Kidney Health
- International Federation of Kidney Foundations
- International Pediatric Nephrology Association (IPNA)
- International Society of Nephrology
- International Society for Peritoneal Dialysis
- Japanese Society for Pediatric Nephrology
- Kidney Care UK
- Latin American Association of Pediatric Nephrology
- Sociedad Latinoamericana de Nefrología e HTA (SLANH)
- The International Diabetes Federation
- The International Society on Thrombosis and Haemostasis
- The World Hypertension League

* The letter to the WHO had 17 signatories (https://www.theisn.org/wp-content/uploads/2025/04/Open-Letter-in-Suppport-for-the-WHO-Resolution-.pdf):
